# The influence of phonon softening on the superconducting critical temperature of Sn nanostructures

**DOI:** 10.1038/s41598-020-62617-4

**Published:** 2020-03-31

**Authors:** Kelly Houben, Johanna K. Jochum, Sebastien Couet, Enric Menéndez, Thomas Picot, Michael Y. Hu, Jiyong Y. Zhao, E. Ercan Alp, André Vantomme, Kristiaan Temst, Margriet J. Van Bael

**Affiliations:** 1 Quantum Solid State Physics, Celestijnenlaan 200D, B-3001 Leuven, Belgium; 2 Instituut voor Kern- en Stralingsfysica, Celestijnenlaan 200 D, B-3001 Leuven, Belgium; 3 Advanced Photon Source, Argonne National Laboratory, Argonne, Illinois 60439 USA

**Keywords:** Magnetic properties and materials, Superconducting properties and materials

## Abstract

The increase in superconducting transition temperature (T_*C*_) of Sn nanostructures in comparison to bulk, was studied. Changes in the phonon density of states (PDOS) of the weakly coupled superconductor Sn were analyzed and correlated with the increase in T_*C*_ measured by magnetometry. The PDOS of all nanostructured samples shows a slightly increased number of low-energy phonon modes and a strong decrease in the number of high-energy phonon modes in comparison to the bulk Sn PDOS. The phonon densities of states, which were determined previously using nuclear resonant inelastic X-ray scattering, were used to calculate the superconducting transition temperature using the Allen-Dynes-McMillan (ADMM) formalism. Both the calculated as well as the experimentally determined values of T_*C*_ show an increase compared to the bulk superconducting transition temperature. The good agreement between these values indicates that phonon softening has a major influence on the superconducting transition temperature of Sn nanostructures. The influence of electron confinement effects appears to be minor in these systems.

## Introduction

In conventional superconductors phonons bind the electrons into Cooper pairs below a critical temperature (T_*C*_). Hence, the spectrum of lattice vibrations, described by the phonon density of states (PDOS), plays a crucial role in conventional superconductivity.Tin is known in two stable phases: Below 285 K, *α*-Sn a semiconductor with quasi zero bandgap and a diamond cubic structure is the stable phase. Above 285 K, Sn crystallises into the body-centred-tetragonal *β*-Sn phase. Due to the low transformation rate from the *β*-Sn phase to the *α*-Sn phase, the *β*-Sn phase can be stabilised at low temperatures where it becomes superconducting below 3.72 K^1^. It belongs to the group of weakly-coupled superconductors (*λ* < 1). We recently studied the *α*-Sn to *β*-Sn transition in thin films through the observation of the PDOS^2^.

When reducing the dimensions of weakly-coupled superconductors (*e.g*. Al, In, Sn *etc*), T_*C*_ is found to increase. For Sn nanostructures an increase in T_*C*_ of up to 10% has been observed^3–10^. The mechanism of this T_*C*_ enhancement is not well understood and is suggested to be caused by changes in the phonon density of states^3,5,11– 14^, changes in the electron density of states^15–23^ or a combination of these effects^24–30^.

In Sn nanowires and nanoparticles the increase in T_*C*_ was attributed to electron confinement effects^6,7,21,23^, whereas in granular films, the origin of the increase in T_*C*_ was speculated to be a change in the phonon density of states^5,12^. In our recent work we could explain the increase in *λ* and T_*C*_ of Sn nanowires with diameters of 18, 35 and 110 nm by phonon softening and an increase in the electron - phonon coupling^31^.

In general, to determine whether an increase in T_*C*_ is caused by the phonon or electron behaviour it is necessary to disentangle their contributions to T_*C*_. However, by measuring *α*^2^(*E*)*g(E)* through tunneling measurements, the electron- and phonon-related effects can not be disentangled. Hence, this approach does not allow a clear determination of the effect of phonon softening on the superconducting properties of nanosized Sn structures.

In this work, we determine the superconducting properties of different Sn nanostructures using the ADMM formalism, from the PDOS extracted earlier^32^ from nuclear resonant inelastic X-ray scattering measurements.

## Results and Discussion

### Structural characterisation

**Figure 1:**
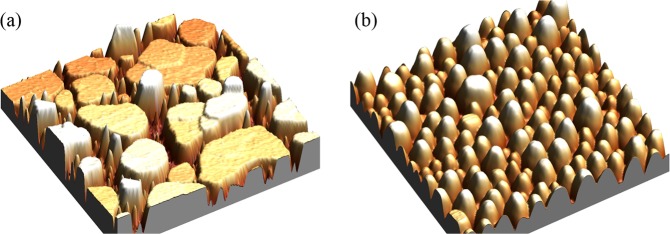
(**a**) AFM image of Sn islands (height scale = 139 nm, nominal thickness = 40 nm, image size = 7 *μ*m  ×  7 *μ*m) ; (**b**) AFM image of Sn cluster-assembled film, (height scale = 53 nm, thickness = 50 nm, image size = 1 *μ*m  ×  1 *μ*m).

The structural characterisation using atomic force microscopy (AFM) and grazing incidence X-ray diffraction (XRD) of the Sn nanostructures shown here, was presented in detail in ref. ^32^. The relevant results of these measurements are summarised in Table 1 and Fig. 1. Figure 1 shows AFM images of the two types of nanostructures highlighting their different morphologies: Fig. 1(a) shows a 53 nm thick, granular cluster-assembled film grown on amorphous SiO_2_ while Fig. 1(b) shows Sn islands, grown on Si(111), with an average island height of 68 ± 17 nm (Fig. 1(b)). To avoid oxidation the nanostructures were capped with Si and Ge respectively. Figure 1 shows clearly that the morphology of the two types of nanostructures is very different. While the Sn islands are textured, with a preferred orientation perpendicular to the substrate, the cluster-assembled film is polycrystalline, consisting of randomly oriented grains formed by coalescence of much smaller deposited clusters. These conclusions are further supported by XRD measurements (not shown). Further details on the sample fabrication and characterisation processes can be found in^32^.

**Table 1:** Characteristics of the investigated samples. For the island samples, the thickness refers to the nominal thickness of Sn (amount of material) deposited on Si(111). For the cluster-assembled film, the thickness is obtained by Rutherford backscattering spectometry. The average crystallite size is obtained from Rietveld refinement of the GIXRD measurements.

Sample	Type	Nominal thickness (nm)	Average island height (nm)	Average *β*-Sn crystallite size (nm)
isl60	nano islands	60	100 ± 21	68 ± 9
isl40	nano islands	40	68 ± 17	54 ± 6
clus46	cluster film	46	—	123 ± 14

### Superconducting properties

**Figure 2:**
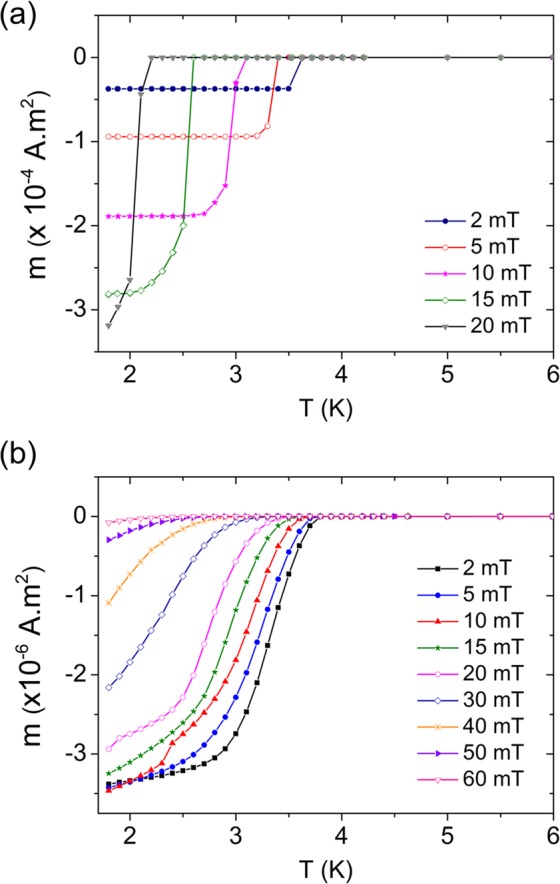
Magnetic moment (*m*) as a function of temperature after zero-field cooling for different applied magnetic fields for: (**a**) ^119^Sn reference foil and (**b**) clus46.

Superconducting quantum interference device (SQUID) magnetometry measurements were carried out to investigate the superconducting properties of the Sn nanostructures. *T*_*C*_ was identified as the onset of the diamagnetic response^33^. The samples were measured in an out-of-plane configuration (*i.e*., applied magnetic field perpendicular to the sample surface). As a bulk reference, a ^119^Sn foil was measured in addition to the nanostructures. The *m(T)* curves of a ^119^Sn reference foil and sample clus46 are shown in Fig. 2 for different applied fields.

**Figure 3:**
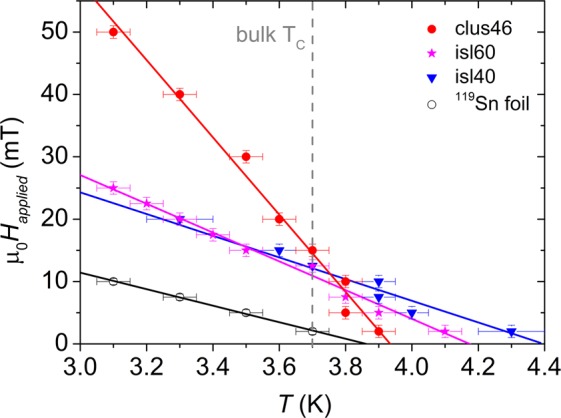
The boundaries of the superconducting phase for the different nanostructures extracted from magnetometry data shown together with the phase boundary of the ^119^Sn foil. The solid lines are linear fits to the measured data points.

For the ^119^Sn foil, a *T*_*C*_ of 3.85 ± 0.05 K was found (see Fig. 2(a)), in good agreement with *T*_*C*_ (= 3.72 K) found for bulk Sn^34^. For sample clus46, an increase in *T*_*C*_ of 5% (see Fig. 2(b)) was found, *T*_*C*_ = 3.93 ± 0.05 K. For isl60 and isl40, an increase in *T*_*C*_ was found as well, *T*_*C*_ = 4.17 ± 0.05 K (11% increase) and 4.38 ± 0.05 K (16% increase) respectively. SQUID measurements of the Sn islands with and without capping layer were compared (not shown), and no difference in the superconducting behaviour was found. The superconducting phase boundaries were constructed from the *m(T)* curves at different magnetic fields, see Fig. 3.

**Figure 4:**
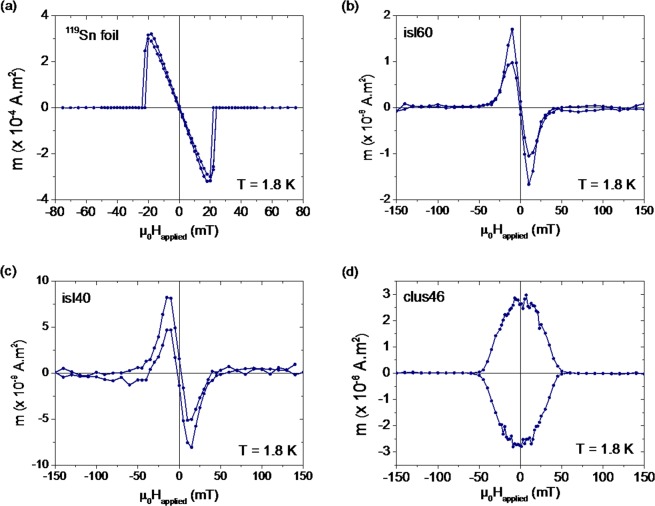
Magnetic moment (*m*) as a function of applied magnetic field at 1.8 K for the different Sn samples.

From the phase boundaries in Fig. 3, the upper critical magnetic field, H_*C*2_, was determined which is listed in Table 2 for all samples. From H_*C*2_ the superconducting coherence length (*ξ*(0)) was calculated. The slope of the superconducting phase boundary for the Sn island samples is larger than for the Sn reference foil, and the slope for the cluster-assembled film is larger still. This increase in slope of the superconducting phase boundary originates in the reduction of mean free path in the nanostructures which is due to the increased scattering and disorder. This disorder leads to a reduced coherence length (*ξ*(0) = 0.855 $\sqrt{{\xi }_{0}l}$^35^) and an increased critical field (*H*_*C*2_(0) = $\frac{{\phi }_{0}}{2\pi \xi {(0)}^{2}}$^35^). Here, *ξ*(*T*) and *ξ*_0_ are the temperature dependent and independent coherence lengths (${\xi }_{0}^{Sn}$ = 230 nm), and *ϕ*_0_ is the magnetic flux quantum (2.0678  × 10^−15^ Wb)^34^). The Sn foil and Sn nanostructures show a linear behaviour of the critical field near *T* ≈ *T*_*C*_ (*H*_*C*2_ = *H*_*C*2_ (0)(1 − *T*/*T*_*C*_)). This is consistent with the behaviour of a dirty type-II superconductor (with mean free path *l* ≪ *ξ*_0_). In addition to the *m(T)* curves, the magnetic moment as a function of applied magnetic field at a fixed temperature, *m*(*H*_*applied*_), was measured for the different samples (Fig. 4).

**Table 2:** Sample characteristics extracted from the superconducting phase boundaries: critical temperature (*T*_*C*_), critical magnetic field (H_*C*2_), coherence length (*ξ*(0)) and mean free path (*l*).

Sample	*T*_*C*_ (K)	*H*_*C*2_ (mT)	*ξ*(0) (nm)	*l* (nm)
^119^Sn foil	3.85 ± 0.05	26 ± 6	113 ± 13	75 ± 17
isl60	4.17 ± 0.05	35 ± 8	97 ± 11	56 ± 12
isl40	4.38 ± 0.05	41 ± 6	90 ± 7	48 ± 7
clus46	3.93 ± 0.05	61 ± 5	74 ± 3	32 ± 3

The *m*(*H*_*applied*_) curve of the Sn foil, measured at 1.8 K, is fully reversible, indicating the absence of magnetic flux pinning (see Fig. 4(a)). Figure 4(b,c) show the results of the *m*(*H*_*applied*_) curves for isl60 and isl40 measured at 1.8 K, respectively. The slight hysteresis observed for these samples indicates a limited pinning of magnetic flux lines in these samples. In Fig. 4(d), the *m*(*H*_*applied*_) curve for sample clus46 at 1.8 K is shown. This curve shows a pronounced hysteresis, and even flux jumps, or flux avalanches, can be distinguished. Before looking into the role of phonons on the observed enhancement of *T*_*C*_, several other mechanisms which could be invoked to explain this *T*_*C*_ increase will be excluded. The isotope effect in Sn is excluded as a potential cause for the observed increase in *T*_*C*_. The former is estimated by:

1 $${M}^{\alpha }{T}_{C}=constant$$

For Sn, *α* ≈  0.46^36^. Natural Sn has a mass of 118.7 amu, and a *T*_*C*_ of 3.72 K. Our enriched ^119^Sn samples have a mass of 119 amu, which would result in a decrease in *T*_*C*_ of 0.1%. The order of magnitude of this effect is by far too small to account for the observed change in *T*_*C*_ and the sign of the effect is opposite of what is observed.

Moreover, due to the fact that the samples studied in this work behave as dirty type-II superconductors, anisotropy of the superconducting gap as a cause for the enhancement of the critical temperature^37–39^ can be excluded as well. The short electron mean free path in dirty type-II superconductors washes out the anisotropy of the superconducting energy gap^38,40^. This effect could otherwise result in an increase in *T*_*C*_ of up to 8%.

Furthermore, it has been predicted that quantum size effects, quantization of the electron spectrum due to dimensional confinement in one direction, also result in an increase of *T*_*C*_^20,21^. Size dependent behaviour of the critical temperature has been attributed to the shape resonance effect^22^, which causes the electron density of states near the Fermi level to change as a function of the sample thickness or nanoparticle size^7,17,23,41,42^. For Sn nanoparticles^23^ quantum size effects have been observed up to a particle size of 20 nm, whereas for Sn nanowires a 1D-like behaviour has been observed up to a nanowire diameter of 40 nm^22^. The crystallite size of the samples which were studied here is 50 nm and larger, which is larger than the typical dimensions of Sn nanostructures for which electron confinement effects have been observed. This suggests that electron confinement effects play a minor role and that the changes in the PDOS are dominating the changes in *T*_*C*_ in the current study.

For the remainder of this work, we will fully concentrate on the role of phonons in the modification of *T*_*C*_ in nanoscale Sn.

### Phonon density of states

**Figure 5:**
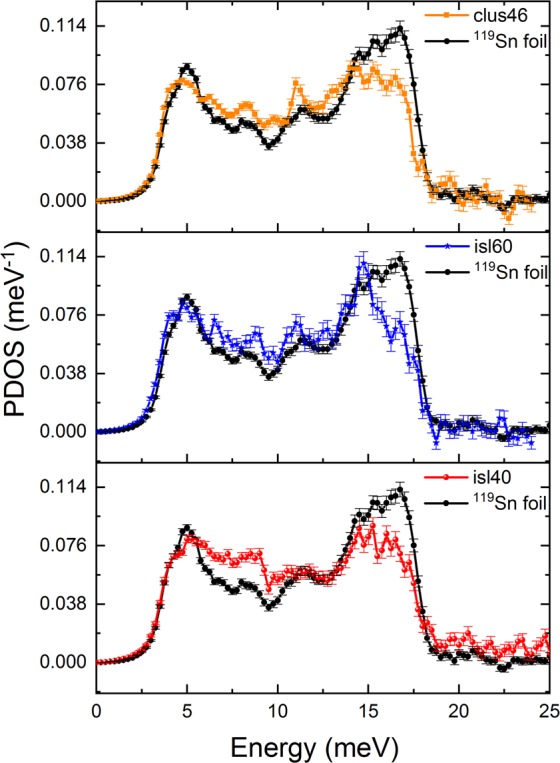
Phonon density of states for the Sn cluster-assembled film and the Sn island samples in comparison to bulk Sn. Data have been reproduced with permission from^32^.

We have studied the PDOS of different Sn nanostructures by nuclear resonant inelastic X-ray scattering^32^ and compared them to the PDOS of a bulk Sn foil. We found a decrease of high-energy phonon modes, a small enhancement of low-energy phonon modes and a general broadening of the PDOS features, see Fig. 5 and ref. ^32^ for a more thorough discussion of the PDOS measurements. These observations can be attributed to the high density of grain boundaries in the investigated nanostructures. Sn atoms at the grain boundaries are in a position of reduced symmetry and coordination. They are held in place by weaker forces than Sn atoms in the bulk, leading to the observed modifications in the PDOS of the nanostructures with respect to bulk.

### Allen-dynes-mcMillan formalism

The previously measured phonon spectra were used to determine the critical superconducting temperature, the characteristic phonon frequency and electron - phonon coupling constant (*λ*) within the Allen-Dynes-McMillan formalism based on the Eliashberg equations^43^. Besides the Coulomb repulsion, also the retarded electron - phonon interaction is explicitly included in the ADMM equation via the electron phonon spectral function *α*^2^(*E*)*g*(*E*), which includes the PDOS and the electron - phonon interaction *α*^2^(*E*)^43^. We have used the following ADMM equation for T_*C*_, a correction to the McMillan expression used for the calculation of T_*C*_ in conventional superconductors^44^.

2 $${T}_{c}=\frac{{f}_{1}{f}_{2}{\omega }_{ln}}{1.2}\cdot \exp \left[\frac{-1.04(1+\lambda )}{\lambda -\mu \ast -0.62\lambda \mu \ast }\right]$$

with μ^*^ the Coulomb pseudopotential which corresponds to a renormalised Coulomb repulsion^43^ and

3 $$\lambda =2\int \frac{{\alpha }^{2}(E)g(E)dE}{E}.$$

Allen and Dynes introduced the correction factors *f*_1_ and *f*_2_ as defined below^43^ to account for strong coupling corrections. These correction factors go towards unity in the weak coupling limit.

4 $${f}_{1}={\left[1+\left(\frac{\lambda }{2.46(1+3.8{\mu }^{\ast })}\right)\right]}^{\frac{1}{3}}$$

5 $${f}_{2}=1+\frac{{\lambda }^{2}\left(\frac{{\langle {\Omega }^{2}\rangle }^{\frac{1}{2}}}{{\omega }_{ln}}-1\right)}{{\lambda }^{2}+\left[1.82\left(1+6.3{\mu }^{\ast }\frac{{\langle {\Omega }^{2}\rangle }^{\frac{1}{2}}}{{\omega }_{ln}}\right)\right]}$$

*f*_2_ depends on the characteristic phonon frequency:

6 $${\langle {\Omega }^{2}\rangle }^{\frac{1}{2}}={\left[\frac{2}{\lambda }\int {\alpha }^{2}(E)g(E)EdE\right]}^{\frac{1}{2}}$$

The logarithmic average frequency used in Eq. (2) is defined as:

7 $${\omega }_{ln}=\exp \left[\frac{2}{\lambda }\int \frac{{\alpha }^{2}(E)g(E){\rm{ln}}\,(E)dE}{E}\right]$$

Also a slightly different analytical expression for T_*C*_ derived from the Eliashberg equations^45^ was applied to calculate T_*C*_. It revealed similar results as will be shown below (see also Supplementary information). In order to calculate *T*_*C*_, ${\langle {\Omega }^{2}\rangle }^{\frac{1}{2}}$, *ω*_*ln*_ using Eqs. (2–7), the electron - phonon interaction, *α*^2^(*E*), and the Coulomb pseudopotential, μ^*^, need to be estimated.

*α*^2^(*E*) *g(E)* was taken from literature^46^, where it was obtained from tunneling measurements on 200 nm thick granular Sn films on glass substrates. *α*^2^(*E*) *g(E)* is subsequently divided by our experimentally obtained PDOS, *g(E)*, of the reference Sn foil to obtain *α*^2^(*E*). The thus obtained *α*^2^(*E*) is an estimation since *α*^2^(*E*) *g(E)* and the PDOS of the Sn foil were not measured on the same Sn sample. However, no information is available in literature on *α*^2^(*E*) of Sn directly. Nonetheless, this approach, which has been used before^47,48^ to determine *α*^2^(*E*) for Nb_3_Sn, provides the best available estimation for *α*^2^(*E*).

**Figure 6:**
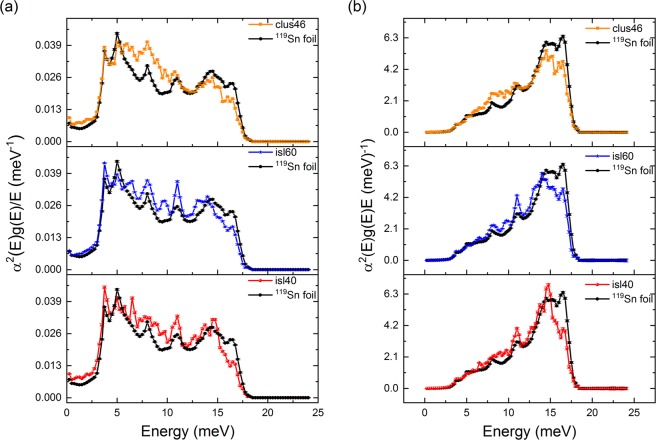
(**a**) The phonon density of states was combined with *α*^2^(*E*) to obtain *α*^2^(*E*) *g(E)*/*E* for the Sn cluster-assembled film and the island samples in comparison to bulk Sn. (**b**) The phonon density of states was combined with *α*^2^(*E*) and replotted as *α*^2^(*E*) *g(E)E* for the different Sn nanostructures and the ^119^Sn reference foil.

In Fig. 6  *α*^2^(*E*)*g(E)*/*E* and *α*^2^(*E*)*g(E)E* are shown, which are integrated for the calculation of *λ* and ${\langle {\Omega }^{2}\rangle }^{1/2}$ respectively (see Table 3). The effect of phonon softening can be seen from the larger values of these functions at low energies and their reduced values at high energies compared to bulk Sn.

**Table 3:** *λ*,  ${\langle {\Omega }^{2}\rangle }^{1/2}$, ∫*g*(*E*)*EdE*, *ω*_*ln*_, ${T}_{C,cal}^{ADMM}$ calculated for all samples, using μ^*^ = 0.117 and ${T}_{C,cal}^{E}$ calculated for all samples, using μ^*^ = 0.119. The last column shows the experimentally obtained critical temperature, *T*_*C*,*exp*_ *(the error on *T*_*C*,*exp*_ is 0.05 K for all samples and is determined by the temperature stability of the SQUID magnetometer, the errors indicated for the other quantities are based on the statistical error of the PDOS).

Sample	*λ*	${{\boldsymbol{\langle }}{{\boldsymbol{\Omega }}}^{{\bf{2}}}{\boldsymbol{\rangle }}}^{{\bf{1}}{\boldsymbol{/}}{\bf{2}}}$	∫*g*(*E*)*EdE*	*ω*_*ln*_	${{\boldsymbol{T}}}_{{\boldsymbol{C}}{\boldsymbol{,}}{\boldsymbol{c}}{\boldsymbol{a}}{\boldsymbol{l}}}^{{\boldsymbol{A}}{\boldsymbol{D}}{\boldsymbol{M}}{\boldsymbol{M}}}$	${{\boldsymbol{T}}}_{{\boldsymbol{C}}{\boldsymbol{,}}{\boldsymbol{c}}{\boldsymbol{a}}{\boldsymbol{l}}}^{{\boldsymbol{E}}}$	*T*_*C*,exp_*
		(meV)	(meV)	(meV)	(K)	(K)	(K)
^119^Sn foil	0.76 ± 0.04	10.4 ± 0.2	11.62 ± 0.03	8.1 ± 0.2	3.7 ± 0.2	3.7 ± 0.2	3.85
isl60	0.82 ± 0.03	9.9 ± 0.3	10.82 ± 0.04	7.8 ± 0.2	4.1 ± 0.2	4.2 ± 0.2	4.17
isl40	0.84 ± 0.06	9.8 ± 0.4	10.74 ± 0.05	7.6 ± 0.3	4.3 ± 0.4	4.4 ± 0.3	4.38
clus46	0.82 ± 0.04	9.8 ± 0.3	10.36 ± 0.03	7.6 ± 0.2	4.1 ± 0.2	4.1 ± 0.2	3.93

The only unknown which is left at this point in Eq. (2) is the Coulomb pseudopotential μ^*^. For bulk Sn, *T*_*C*_ is known to be 3.72 K. By equating *T*_*C*_ in Eq. (2) to 3.72 K and using the phonon spectrum of bulk Sn, μ^*^ is estimated to be 0.117. The value of μ^*^ agrees well with values reported in literature^5,45,49^ and it is known that for most metals, μ^*^ should be ≤0.2^50^.

These empirical values for μ^*^ and *α*^2^(*E*) were fixed (μ^*^ and *α*^2^(*E*) were taken to have the same values for all Sn samples) and were used to calculate *T*_*C*,*cal*_ using the experimentally determined phonon spectrum of the corresponding sample. In Table 3, *λ*, ${\left\langle {\Omega }^{2}\right\rangle }^{1/2}$, *ω*_*ln*_, *T*_*C*,*cal*_ and *T*_*C*,*exp*_ are listed for all samples. Table 3 shows the obtained values for *λ*, ${\left\langle {\Omega }^{2}\right\rangle }^{1/2}$ and *ω*_*ln*_ as well as the intuitive average phonon frequency (energy) obtained by integrating *g*(*E*)*EdE* without taking the electron phonon coupling into account. It can be seen that this average frequency (energy) is significantly reduced (7–11%) for the nanostructured samples compared to the bulk Sn foil. Furthermore, the characteristic phonon frequencies ${\langle {\Omega }^{2}\rangle }^{\frac{1}{2}}$ and *ω*_*ln*_ for all the nanostructured samples are up to 6% lower than for the bulk Sn foil, which indicates that the phonon spectra of all nanostructures are softer than that of the bulk Sn foil. This is indeed expected from the high density of grain boundaries present in all Sn nanostructures, which causes a decrease in the high-energy phonon modes and a slight enhancement in the low-energy phonon modes. This clear trend of phonon softening goes along with an increase of the electron - phonon coupling parameter *λ* of up to 10 % in the nanostructured samples. Table 3 also displays the experimental and calculated (using Eq. (2)) critical temperatures T_*C*,*exp*_ and T_*C*,*calc*_. Clearly, T_*C*,*calc*_ in the nanostructured samples is enhanced up to 19% compared to the bulk sample as a result of the modifications in the PDOS, i. e. due to phonon softening.

From the agreement between *T*_*C*,*cal*_ and *T*_*C*,*exp*_ ($\left|{T}_{C,cal}-{T}_{C,exp}\right|$ <  5%) it was concluded that the phonon softening observed for the nanostructures is the main contribution to the increase in *T*_*C*_. The contribution of electron confinement effects to the change in *T*_*C*_ is minimal and of secondary importance.

**Figure 7:**
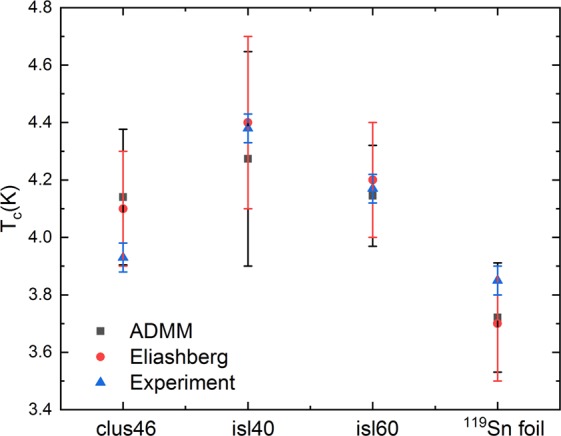
Comparison of T_*C*,*exp*_ and T_*C*,*calc*_ for the ADMM as well as the Eliashberg formalism.

To further confirm these results T_*C*_ was also calculated using an analytical experssion based on the Eliashberg formalism shown by Kresin^45^ (see Supplementary information). We further refer to these results as the Eliashberg results and compare them with the T_*C*_ obtained using the ADMM formalism. Both these formalisms are extensions of the original Bardeen - Cooper - Schrieffer theory for a larger range of electron - phonon coupling strengths (*λ*) and are expected to give comparable results for the low *λ* found for Sn. We indeed find the same values of T_*C*_ for all samples within error bars independent of the used expression as can be seen in Fig. 7 (T${}_{C,cal}^{ADMM}$, for the ADMM results and T${}_{C,cal}^{E}$ for the Eliashberg results).

The dominance of phonon softening in the increase of T_*C*_ is different to what has been observed previously in the case of Nb_3_Sn thin films^48^. For Nb_3_Sn, a strong coupling superconductor, the phonon induced effects were found to induce a slight decrease in *T*_*C*_ with decreasing film thickness. This phonon-related decrease in *T*_*C*_ was found to be only a small fraction of the experimentally observed decrease in *T*_*C*_, the main cause being electron confinement effects. This difference in behaviour of *T*_*C*_ of Nb_3_Sn films and Sn nanostructures can be explained by the fact that Nb_3_Sn is a strongly coupled superconductor, while Sn is a weakly coupled superconductor.

## Conclusions

We determined the characteristic phonon frequency (${\langle {\Omega }^{2}\rangle }^{\frac{1}{2}}$), the electron - phonon coupling strength (*λ*) as well as the superconducting critical temperature *T*_*C*_ from the PDOS of nanostructured Sn samples using the Allen-Dynes-McMillan^43^ and Eliashberg^45^ formalisms. The PDOS of the samples were experimentally obtained in our previous work using nuclear resonant inelastic X-ray scattering and showed clear phonon softening in the nanostructured samples^32^. The softening of the characteristic phonon frequency is accompanied by an increase of the electron - phonon coupling strength (*λ*). We compared the calculated values of T_*C*_ to SQUID magnetometry measurements on the same samples. Both methods show an increase in *T*_*C*_ for the nanostructures. The possible causes of this increase in *T*_*C*_ was discussed. The isotope effect, the anisotropy of the superconducting gap as well as quantum size effects could be excluded. Electron confinement effects were considered unlikely and playing only a secondary role in the enhancement of *T*_*C*_ observed here. The good agreement between the experimentally obtained critical temperature of the Sn nanostructures and the *T*_*C*_ calculated based on the measured PDOS confirms that phonon softening effects play the dominate role in the observed enhancement of *T*_*C*_.

## Methods

Details on the growth and structural characterization of the Sn nanostructures can be found in ref. ^32^. The Sn islands were grown using molecular beam epitaxy on Si(111) substrates. The Sn cluster-assembled film was grown on a SiO_2_ substrate using a laser-vaporization cluster source^51^. The AFM images were recorded using a Nanowizard 3 system (JPK, Germany) and a Multimode 8 system (Bruker, USA) and processed using WSxM^52^. The superconducting behaviour of the different Sn samples was probed by SQUID magnetization measurements (LOT-Quantum Design, MPMS-XL). The phonon density of states of all Sn samples was measured using nuclear resonant inelastic X-ray scattering^53–55^ at sector 3-ID of the Advanced Photon Source (Argonne National Laboratory, USA). Measurements were carried out at 35 ± 20 K in a grazing incidence geometry. The data were analyzed using the PHOENIX software^56^. A more elaborate discussion of the nuclear resonant inelastic X-ray scattering measurements can be found elsewhere^32^.

## Supplementary information

Supplementary Information.
