# Supplementary material for: The influence of phonon softening on the superconducting critical temperature of Sn nanostructures
Source: Sci Rep. 2020 Mar 31;10:5729. doi: 10.1038/s41598-020-62617-4 (PMC7109077; doi:10.1038/s41598-020-62617-4)
Supplement: Supplementary file 1 — Supplementary Information. [file 41598_2020_62617_MOESM1_ESM.pdf]

The influence of phonon softening on the superconducting critical temperature of Sn nanostructures

Kelly Houben[1], Johanna K. Jochum[1], Sebastien Couet[2], Enric Menéndez[2], Thomas Picot[1], Michael Y. Hu[3], Jiyong Y. Zhao[3], Esen E. Alp[3], André Vantomme[2], Kristiaan Temst[2] & Margriet J. Van Bael[1]

[1]*Quantum Solid State Physics, Celestijnenlaan 200D, B-3001 Leuven, Belgium*

[2]*Instituut voor Kern- en Stralingsfysica, Celestijnenlaan 200 D, B-3001 Leuven, Belgium*

[3]*Advanced Photon Source, Argonne National Laboratory, Argonne, Illinois, 60439, USA*

1 Supplementary Information

Eliashberg formalism: The Eliashberg formalism is the natural development of the BCS theory to include retardation effects, due to the slow response of the phonons in comparison to the electron response (1; 2).

The phonon spectrum is linked to the superconducting transition temperature T_C by the following relationship (3):

$$T_C = \frac{0.25}{\left(e^{2/\lambda_{eff}} - 1\right)^{\frac{1}{2}}} \langle \Omega^2 \rangle^{\frac{1}{2}} \tag{1}$$

The characteristic phonon frequency $\langle \Omega^2 \rangle^{\frac{1}{2}}$ and the electron - phonon coupling constant λ

are defined analoguously to the ADMM formalism (see Eqs. 6 and 3 respectively).

λ_{eff} is the effective coupling constant, given by the following expression:

$$\lambda_{eff} = \frac{\lambda - \mu^*}{1 + 2\mu^* + \lambda\mu^* t(\lambda)}, \tag{2}$$

where μ^*, is the Coulomb pseudopotential of the material and corresponds to a renormalized Coulomb repulsion. t(λ) is a universal function given by:

$$t(\lambda) = 1.5e^{-0.28\lambda} \tag{3}$$

Eq. 1 holds for any strength of the electron phonon coupling (λ) (3). $\alpha^2(E)$ as well as μ^* were determined in the same manner as for the ADMM formalism. A small difference in μ^* was found for the two formalisms: μ^*_{ADMM} = 0.117 while μ^*_E = 0.119. And while, as shown in Fig. **??**, the values for T_C do not perfectly coincide, the discrepancy between the calculated values is small and lies well within the errorbars of each other.

1. G. M. Eliashberg, "Interactions between electrons and lattice vibrations in a superconductor," *Sov. Phys.-JETP (Engl. Transl.);(United States)*, vol. 11, no. 3, 1960.

2. F. Marsiglio and J. P. Carbotte, "Electron-phonon superconductivity," in *Superconductivity*, pp. 73–162, Springer, 2008.

3. V. Z. Kresin, “On the critical temperature for any strength of the electron-phonon coupling,” *Physics Letters A*, vol. 122, no. 8, pp. 434–438, 1987.
